# Impact of Groundwater Salinity on Bioremediation Enhanced by Micro-Nano Bubbles

**DOI:** 10.3390/ma6093676

**Published:** 2013-08-23

**Authors:** Hengzhen Li, Liming Hu, Zhiran Xia

**Affiliations:** State Key Laboratory of Hydro-Science and Engineering, Department of Hydraulic Engineering, Tsinghua University, Beijing 100084, China; E-Mails: lihengzhen09@gmail.com (H.L.); xzr09@mails.tsinghua.edu.cn (Z.X.)

**Keywords:** micro-nano bubbles, groundwater bioremediation, salinity, size distribution, oxygen transfer, zeta potential, permeability

## Abstract

Micro-nano bubbles (MNBs) technology has shown great potential in groundwater bioremediation because of their large specific surface area, negatively charged surface, long stagnation, high oxygen transfer efficiency, *etc*. Groundwater salinity, which varies from sites due to different geological and environmental conditions, has a strong impact on the bioremediation effect. However, the groundwater salinity effect on MNBs’ behavior has not been reported. In this study, the size distribution, oxygen transfer efficiency and zeta potential of MNBs was investigated in different salt concentrations. In addition, the permeability of MNBs’ water through sand in different salt concentrations was studied. The results showed that water salinity has no influence on bubble size distribution during MNBs generation. MNBs could greatly enhance the oxygen transfer efficiency from inner bubbles to outer water, which may greatly enhance aerobic bioremediation. However, the enhancement varied depending on salt concentration. 0.7 g/L was found to be the optimal salt concentration to transfer oxygen. Moreover, MNBs in water salinity of 0.7 g/L had the minimum zeta potential. The correlation of zeta potential and mass transfer was discussed. The hydraulic conductivities of sand were similar for MNBs water with different salt concentrations. The results suggested that salinity had a great influence on MNBs performance, and groundwater salinity should be taken into careful consideration in applying MNBs technology to the enhancement of bioremediation.

## 1. Introduction

*In situ* groundwater bioremediation is one of the most common and environment-friendly methods to remediate polluted groundwater [1,2,3,4,5]. The activity of microorganisms is related to dissolved oxygen availability, groundwater salinity, nutrients, pH value, *etc.* [6,7,8,9,10,11]. The groundwater salinity varies owing to different groundwater mobility, precipitation and soil and/or rock solubility. Sea water invasion in offshore area and human activities will also influence the groundwater salinity [12]. The salinity impact on bioremediation has been studied since the 1990s [6,13,14,15,16,17], and is considered to be an important factor in influencing bioremediation.

Micro-nano bubbles (MNBs) technology has attracted much interest in bio-related areas such as bioremediation, aquaculture and plant cultivation [18,19,20]. MNBs are tiny bubbles with diameters of micrometers and nanometers, respectively. Micro bubbles with a 30 μm radius were first found by Turner [21] in water and existed for long time, probably because the surface properties were changed to support the excess inner gas pressure which would slow down the gas diffusion process. Nano bubbles of nitrogen, methane, or argon with radius of 50 nm have a lifetime of more than two weeks [22]. It was found that some nano bubbles can even exist in water for months [23].

MNBs have large specific surface area. It can be calculated that the surface area (*S*) per unit volume (*V*) of a bubble is inversely proportional to the bubble radius (*r*) by using the equations:

(1) $$S=4\pi r^{2}$$

(2) $$V=4/3\pi r^{3}$$

(3) S/V=3/r

Thus, a micro bubble with radius of 1 μm has 1000 times the specific surface area of a conventional bubble with radius of 1 mm.

Bubbles in pure water were negatively charged [24]. The zeta potential measured in water with oxygen MNBs was from −45 mV to −34 mV while air MNBs a little lower which is from −20 mV to −17 mV [19]. The large specific surface area and charged surface enable tiny bubbles to effectively adsorb oppositely charged molecules and/or small particles [25].

Bubble size will influence the gas dissolution process. Smaller bubbles would enhance the gas dissolution process [26]. For MNBs, mass transfer efficiency from inner bubbles to surrounding liquid increases with a decrease in the bubble size and an increase in bubble internal pressure. Therefore, high mass transfer efficiency is expected in MNBs. Consequently, supplying oxygen in the form of MNBs is promising in oxygen-consuming processes like the aerobic bioremediation [27,28].

MNBs have also exhibited some particular biological activities which cannot be illustrated only by the dissolved oxygen enhancement [29,30]. The acceleration of metabolism is possibly related to the free radicals released during the bubble collapse. During the collapse, the ion concentration around the shrinking gas-water interface increases, resulting in the radical generation [31].

Due to the properties mentioned above, MNBs show potential for enhancing bioremediation. However, many properties associated with MNBs are still not clear. This is the first investigation to report on the influence of salinity on MNB performance.

In this study, the salinity impacts on MNBs size distribution, oxygen transfer efficiency, bubble interface zeta potential and hydraulic conductivities of sand with MNBs water were studied. Different salt concentrations were selected to generate different MNBs.

This study is aimed to evaluate the salinity impact on MNBs, for further prediction of the groundwater bioremediation enhancement by MNBs for different site conditions.

## 2. Materials and Methods

### 2.1. Experimental Set-Up

#### 2.1.1. Generation Method

Generation methods have an impact on the MNBs properties. Four categories, which are hydrodynamic, acoustic, optic and particle cavitation, are used to generate MNBs [18]. Compared to other methods, hydrodynamic cavitation is newly developed and has been shown to be more cost-effective and efficient [32].

**Figure 1:**
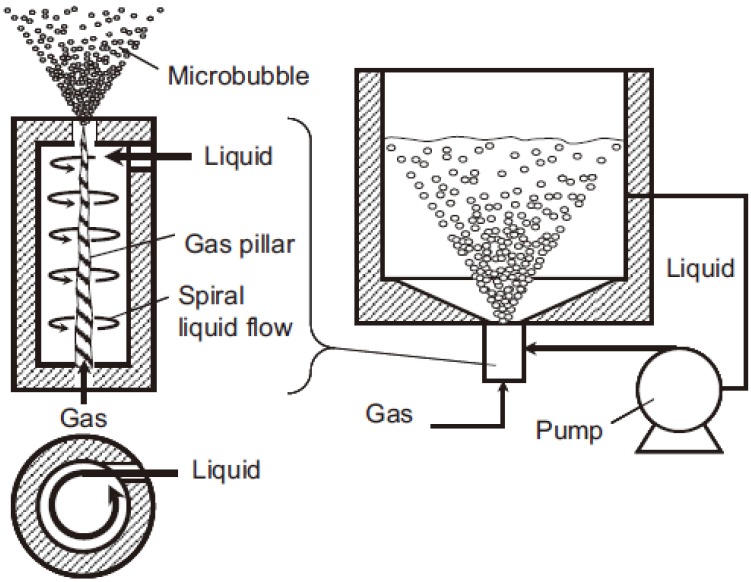
Spiral liquid flow generation method.

The spiral liquid flow type MNBs generation method is one widely-used type of hydrodynamic cavitation [33]. The inner structure of a spiral liquid flow type micro bubble generator is shown in Figure 1 [34].

Gas together with the liquid was injected or absorbed into the cylinder, after which a strong shear force was acted on the liquid to produce spiral liquid flow, forming maelstrom-like cavity in the cylinder. The MNBs were generated in the liquid in this way. The generator used in this study had a gas inlet flow rate of 240 mL/min and water input flow rate of 11 L/min. The output flow rate of MNBs water was 11 L/min.

#### 2.1.2. Bubble Size Distribution Analyzer

The size distribution of MNBs was measured by Malvern Mastersizer LS13320 (Malvern, Inc., Worcestershire, UK). The analyzer was based on laser-light scattering technique, which was proved to be workable for bubble size measurement [35,36]. The measuring range was from 400 nm to 2000 μm.

#### 2.1.3. Dissolved Oxygen Sensor

The dissolved oxygen in water was measured by YSI ProODO meter (Yellow Springs, OH, USA). The meter was optical, luminescent based, calculating the oxygen molecule amount near the meter sensor according to the fluorescence quenching method. The salinity was an input parameter to calculate the dissolved oxygen. The measuring range is from 0 to 50 mg/L (0% to 500% air saturation) with the accuracy of ±1% (0 to 20 mg/L) and ±10% (20 to 50 mg/L).

#### 2.1.4. Zeta Potential Analyzer

The zeta potential of the MNBs was measured by DelsaNano C zeta potential analyzer (Beckman Coulter, Inc., Danvers, MA, USA). The MNBs were irradiated with a laser light (dual 30 mW laser diodes, 658 nm with two scattering angles 15° and 30°) and the scattered light emitted from the bubbles was detected. The frequency of the scattered light was shifted from the incident light in proportion to the speed of the bubbles’ movement. Therefore, the bubbles electrophoretic mobility for calculating zeta potential can be measured.

#### 2.1.5. Permeameter

The permeameter was TST 70 by Nanjing Soil Instrument Factory Co., Ltd. (Jiangsu, China) with diameter of 100 mm and height of 400 mm. The constant hydraulic head was employed for the permeability tests.

### 2.2. Materials

All the water used in the experiment was ultrapure water produced by a water purification system (Direct Q3, Merck Millipore Ltd., Billerica, MA, USA). The gas used for the micro-nano bubbles was air. Sodium chloride (NaCl) was added in different concentrations which were 0 g/L, 0.1 g/L, 0.4 g/L, 0.7 g/L, 1 g/L, 3 g/L, 5 g/L, 7 g/L and 9 g/L. In the permeability test, the British standard sand with size ranging from 0.09 mm to 0.15 mm was used. The effective size is 110 μm and average size 140 μm, dry density 1.57 g/cm, porosity 0.405.

### 2.3. Experimental Procedure

MNBs were generated in different salt concentrations, shown in Table 1. In each test group, the bubbles size distribution, the oxygen transfer rate, the zeta potential and the hydraulic conductivities of sand with MNBs water were tested.

The bubble generation apparatus started to generate MNBs in a water tank with 11 L water in it for 15 min (the water would be cycled for 15 times) and then stopped. After generation, water samples were then immediately collected for particle size analysis. Three water samples were collected and five replications were conducted for each sample.

For the dissolved oxygen measurement, the dissolved oxygen (DO) sensor started to record data when the generation began, and until DO became 100% (saturated). The initial dissolved oxygen value in water was controlled at about 0 mg/L using water vacuum-pumping system. The recording time interval was selected 1 min for the first 30 min and 15 min for the rest time.

**Table 1:** Different test groups for generating MNBs.

Test group	Water volume	Temperature (°C)	Salinity (g/L)
1	11 L ultrapure water	20	0
2	11 L ultrapure water	20	0.1
3	11 L ultrapure water	20	0.4
4	11 L ultrapure water	20	0.7
5	11 L ultrapure water	20	1.0
6	11 L ultrapure water	20	3.0
7	11 L ultrapure water	20	5.0
8	11 L ultrapure water	20	7.0
9	11 L ultrapure water	20	9.0

For the zeta potential measurement, bubble water samples were periodically taken after stable generation. The minimum sample volume was 0.7 mL. Five samples in the same condition would be collected for the zeta potential measurement. For each group the test was repeated 3 times to get an average value.

For the permeability test, the same soil sample was made in each test group. Moreover, air free water went through the sand sample to obtain the saturated permeability. The permeability of micro-nano bubble water through sand was measured following SL237-1999 instructions (the Chinese Hydraulic Ministry). Hydraulic conductivity for each soil sample was obtained by averaging three test results with different hydraulic heads.

All the tests mentioned above were carried out at a temperature of 20 °C.

## 3. Results and Discussion

### 3.1. Bubble Size Distribution

After the MNBs were generated, the water was filled with bubbles and was milky-like. The average bubble sizes of every test group and standard deviations were shown in Table 2. The average bubble sizes in different test groups were almost the same. The low standard deviation indicated small data variation from the average. Thus, the water salinity had no obvious influence on bubble size.

The average diameter of MNBs was around 50 μm, much smaller than the sand pore size. Therefore, these tiny bubbles could transport with groundwater and get into micro areas to enhance the bioremediation effect on a larger scale.

**Table 2:** Average MNBs size of different test groups.

Test group	Average bubble diameter (μm)	Standard deviation (μm)
1	33.44	13.30
2	45.78	14.97
3	52.01	11.13
4	55.38	7.32
5	59.49	12.13
6	54.62	14.05
7	53.61	15.07
8	55.54	14.07
9	57.01	18.00

### 3.2. Oxygen Transfer Efficiency

**Figure 2:**
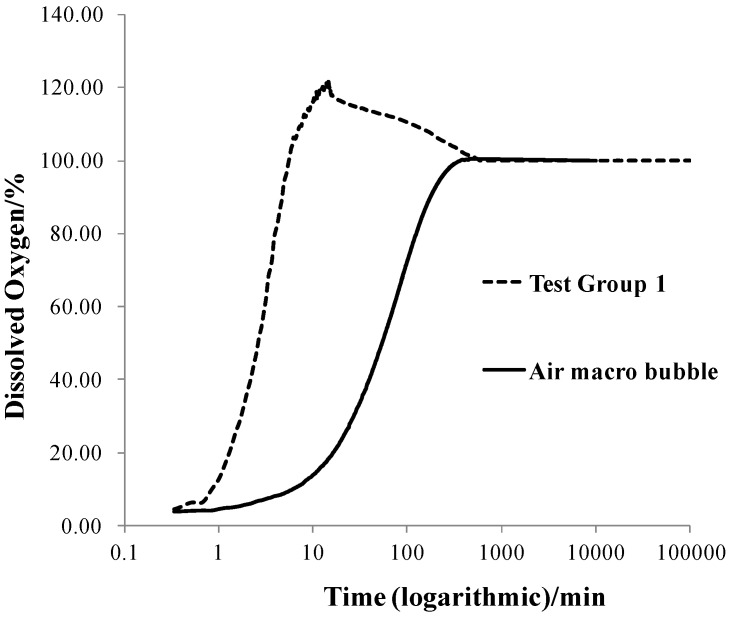
Dissolved oxygen changes with time of test group 1 and macro bubbles.

The dissolved oxygen in different test groups was shown in the form of percentage over saturation value. Figure 2 shows the dissolved oxygen changes with time of test group 1, compared to that of air macro bubbles in the same water condition. It can be seen that the peak value of dissolved oxygen is 120%, much higher than air macro bubbles (100%), which indicates high oxygen transfer efficiency.

**Figure 3:**
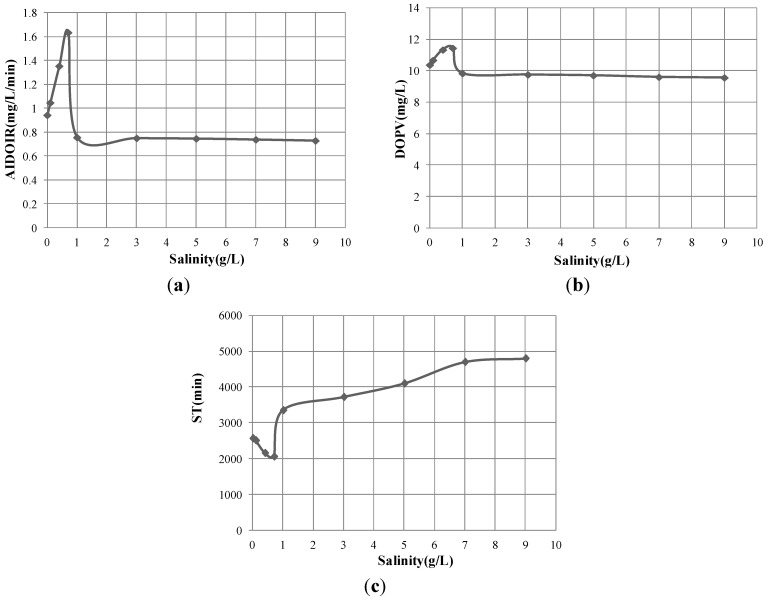
Impact of salinity on oxygen transfer process. (**a**) AIDOIR in different salinity; (**b**) DOPV in different salinity; and (**c**) ST in different salinity.

The dissolved oxygen of Test Group 1 increased quickly at first and reached a peak value, then slowly decreased to a stable value (100%). Other test groups showed the same pattern. Therefore, to compare the dissolved oxygen changes in different groups, three parameters were defined: the dissolved oxygen peak value (DOPV), the average initial dissolved oxygen increasing rate (AIDOIR) which equals to the dissolved oxygen increment (from initial value to peak value) divided by time, and the stagnation time (ST) which means the time dissolved oxygen decreases from the peak value to a stable value (100%). These three parameters show the extent and persistence of dissolve oxygen enhancement. The three parameters in every group were shown in Figure 3.

In Figure 3, salinity 0.7 g/L was a turning point for all three parameters. AIDOIR and DOPV reached the maximum value, 1.636 mg/L/min and 11.45 mg/L respectively, while ST reached the minimum value (2060 min) at salinity 0.7 g/L. The mechanism is discussed in Section 3.4.

It can also be seen that when the salinity is higher than 7 g/L, the salinity influence is slight.

Figure 3 also shows that MNBs could largely enhance the dissolved oxygen by increasing the AIDOIR and DOPV. Besides, the ST is relatively long (more than 34 h), extending the bioremediation enhancement time.

### 3.3. Zeta Potential

**Figure 4:**
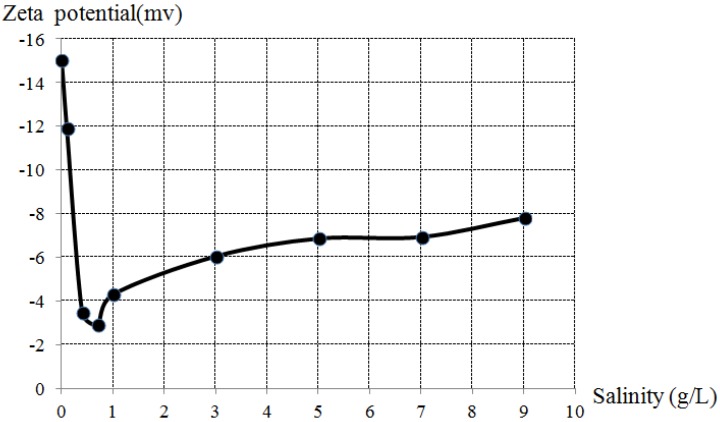
Zeta potentials of MNBs in different salinity.

The effect of salinity on the zeta potential of MNBs is shown in Figure 4. The zeta potential at each salinity was averaged by five samples results. The standard deviations were all below 2 mV, which indicated all the data were close to the average and the test results were convincible. The same trend as ST was found in zeta potential. When the salinity was below 0.7 g/L, the zeta potential decreased as salinity increased. However, after the salinity reached 0.7 g/L, the zeta potential gradually increased. The relation between zeta potential and dissolved oxygen was clear and the possible reason for this trend is discussed in Section 3.4.

MNBs in different salinity were negatively charged, enabling MNBs to effectively adsorb oppositely charged microorganism. In addition, when the bubble collapses, the ion concentration around the gas-water interface increases, resulting in the radical generation, which may be related to the biological activity.

### 3.4. Discussion

The possible charging mechanism can be stated by hydration energy theory. The hydration energy is the energy released upon attachment of water molecules to ions. The hydration energy of OH^−^ is −489 kJ/mol, much lower than that of H^+^ (−1127 kJ/mol), meaning that OH^−^ is more inclined to stay at the gas-water interface. Therefore, when NaCl was added in MNBs water, Na^+^ is apt to be adsorbed as counter-ion for the excess of OH^−^ at the interface. When the salinity is 0.7 g/L, an equilibrium state at the interface was achieved (zeta potential reached a minimum value −2.9 mV). Afterwards, Cl^−^ was more easily adsorbed at the interface than Na^+^ because Cl^−^ had a slightly lower hydration energy (−317 kJ/mol) than Na^+^ (−406 kJ/mol), resulting in the increase in zeta potential.

Lower zeta potential leads to higher AIDOIR and DOPV, and shorter ST. When the zeta potential of MNBs is relatively lower, the Coulomb repulsion force between bubbles is smaller. Therefore, MNBs get closer to each other. In a macro scale, the amount of MNBs in unit water volume is larger, causing greater dissolved oxygen enhancement. For ST, lower zeta potential makes the MNBs more likely coalesce, resulting in less ST. In addition, as mentioned above, the zeta potential is related to the amount of ions at the interface. Lower zeta potential indicates fewer ions, which will exert less resistance to gas diffusion. For these two reasons, lower zeta potential leads to shorter ST.

### 3.5. Permeability Results

The hydraulic conductivities of sand with different salinities and standard deviations are shown in Table 3. The standard deviations showed acceptable variation of different hydraulic conductivity results from the average and proved the results reliable. In Table 3, no obvious difference was shown among different salinities. Furthermore, the hydraulic conductivity of sand with air free water (without NaCl) was 6.40 × 10^−6^ m/s, nearly the same as that of MNBs water. Therefore, the water salinity had no impact on the MNBs water permeability of sand. Moreover, the MNBs will not reduce the groundwater mobility.

Then the MNBs water flow distribution in groundwater can be described as that of air free water.

**Table 3:** Hydraulic conductivities of sand with MNBs water in different salinities.

Salinity (g/L)	Hydraulic conductivity (10^−6^ m/s)	Standard deviation (10^−6^ m/s)
0.0	5.00	2.16
0.1	4.84	0.73
0.4	4.80	1.19
0.7	4.76	2.28
1.0	4.61	0.41
3.0	4.53	0.33
5.0	4.72	1.76
7.0	4.56	1.35
9.0	4.66	3.07

## 4. Conclusions

In this paper, the possible enhancement of groundwater bioremediation by MNBs was stated and the salinity influence on MNBs performance was studied. The following main conclusions can be drawn:

1. Water salinity had no influence on MNBs size.
2. The greater the dissolved oxygen enhancement, the shorter the bubble stagnation time was achieved at the salinity of 0.7 g/L compared to those at other salinities.
3. The lowest zeta potential value was obtained as –2.9 mV at the salinity of 0.7 g/L.
4. Water salinity had no impact on the MNBs water permeability of sand.

The results also suggest that micro-nano bubbles would greatly enhance bioremediation by accelerating oxygen transfer process. Groundwater salinity has a significant influence on MNBs’ properties, and should be taken into consideration when MNBs technology is applied.
